# Reviewer acknowledgement 2013

**DOI:** 10.1186/2051-4190-24-2

**Published:** 2014-02-07

**Authors:** Roger Mieusset

**Affiliations:** CHU Hôpital Paule de Viguier, Toulouse, France

## Abstract

The Editors of Basic and Clinical Andrology would like to thank all our reviewers who have contributed to the journal in Volume 23 (2013).

**Robert Aitken**

Australia

**Deepa Bhartiya**

India

**Florence Boitrelle**

France

**Rafael Boscolo-Berto**

Italy

**Nancy Brackett**

United States of America

**Geoffry De Luliis**

Australia

**Christelle Delalande**

France

**Stéphane Droupy**

France

**Barbara Ferreira da Silva**

Brazil

**Jose Franco Jr**

Brazil

**Francois Giuliano**

France

**Dorothy Greenfeld**

United States of America

**Darren Griffin**

United Kingdom

**Björn Heindryckx**

Belgium

**Takashi Ljiri**

Japan

**David Jareño Martinez**

Belgium

**Clement Jimenez**

France

**Meritxell Jodar Bifet**

United States of America

**Nicolas Kalfa**

France

**Erdal Karaoz**

Turkey

**David Katz**

United States of America

**Yves Menezo**

France

**Ralph Meyer**

United States of America

**Roberto Molina**

Spain

**Gerard Morton**

Canada

**Joao Batista Oliveira**

Brazil

**Thonneau Patrick**

France

**Pierre Ray**

France

**Rodolfo Rey**

Argentina

**Mai Sarraj**

Australia

**Lone Schmidt**

Denmark

**Richard Sharpe**

United Kingdom

**Robert Sullivan**

Canada

**Nicolas Thiounn**

France

**Patrick Thonneau**

Tunisia

**Aminata Toure**

France

**Frank Tüttelmann**

Germany

**Mitchell Valerie**

France

**Francois Vialard**

France

**Chad Wayne**

United States of America

